# Exploring the somatic mutational landscape of ovarian cancer in Estonia

**DOI:** 10.2340/1651-226X.2026.45612

**Published:** 2026-05-18

**Authors:** Mikk Tooming, Kadri Rekker, Kadri Toome, Laura Roht, Piret Laidre, Olga Fjodorova, Ustina Šamarina, Sander Pajusalu, Mihkel Ilisson, Pilleriin Peets, Tõnu Jõgi, Karin Klaup, Kristiina Ojamaa, Eva-Maria Niine-Roolaht, Piret Kaarde, Elen Vettus, Jüri Piirsoo, Helen Vahar, Katrin Õunap, Tiina Kahre

**Affiliations:** a Department of Genetics and Personalized Medicine, Institute of Clinical Medicine, University of Tartu, Tartu, Estonia; b Department of Laboratory Genetics, Genetics and Personalized Medicine Clinic, Tartu University Hospital, Tartu, Estonia; c Department of Clinical Genetics, Genetics and Personalized Medicine Clinic, Tartu University Hospital, Tartu, Estonia; d Chair of Analytical Chemistry, Institute of Chemistry, University of Tartu, Tartu, Estonia; e Haematology and Oncology Clinic, Tartu University Hospital, Tartu, Estonia; f Oncology and Haematology Clinic, North Estonian Medical Center, Tallinn, Estonia; g Centre of Oncology, East Tallinn Central Hospital, Tallinn, Estonia; h Gynaecology Department, West Tallinn Central Hospital, Tallinn, Estonia

**Keywords:** ovarian neoplasms, high-throughput nucleotide sequencing, genetic testing

## Abstract

**Background and purpose:**

This retrospective study provides Estonia’s first nationwide overview of pathogenic and likely pathogenic variants in ovarian cancer (OC) using routine tumor molecular profiling. The aim was to characterize the mutational landscape, identify clinically actionable alterations, and evaluate the integration of germline testing.

**Patient/material and methods:**

A total of 339 OC tumor samples underwent next‑generation sequencing-based profiling. Clinical characteristics, histological subtype, and prior germline testing were recorded. Variants were classified according to clinical‑actionability levels, and homologous recombination deficiency (HRD) was assessed in high‑grade serous OC cases.

**Results:**

The mean age at diagnosis was 62.4 ± 12.5 years, and high‑grade serous OC accounted for 82.0% of cases. Clinically actionable alterations (OncoKB Level 1&2) were detected in 37.5% of patients, most frequently involving *BRCA1*, *BRCA2*, *KRAS*, and *BRAF*. Additional therapeutic targets were identified across diverse biomarkers. HRD was present in 53.2% of tested high‑grade serous tumors. Germline testing had been performed in 41.9% of patients, revealing pathogenic or likely pathogenic variants in 22.5%, predominantly in *BRCA1* and *BRCA2*.

**Interpretation:**

This study outlines the mutational spectrum of OC in Estonia and demonstrates a substantial prevalence of actionable alterations and HRD. The findings highlight the value of comprehensive tumor profiling to support precision‑medicine approaches and improve individualized patient management in Estonia.

## Introduction

According to the World Health Organization (WHO), ovarian cancer (OC) is the seventh most common cancer in women [1]. In Estonia, on average, ~140 people develop OC per year, according to the Estonian National Institute of Health Development, Health Statistics and Health Research Database [2]. The majority of newly diagnosed OC patients present with advanced-stage disease, which can contribute to poor survival outcomes despite advances in surgical and systemic treatment [3]. Cancer development is a complex, multistep process driven by pathogenic/likely pathogenic (PV) in genes that regulate cell growth and/or DNA repair mechanisms [4, 5]. These PV can be of germline or somatic origin, and understanding their contribution is essential for elucidating the mechanisms of inherited and acquired diseases [5]. Current clinical guidelines recommend offering genetic testing (GT) for risk evaluation to all women diagnosed with OC [5, 6]. Identifying germline PV can also prompt cascade screening for at-risk relatives. Germline variants, inherited in an autosomal dominant pattern [7], markedly increase the lifetime risk of developing specific cancers, frequently manifesting at an early age. For example, carriers of germline *BRCA1* PV have an estimated 58% lifetime risk of OC, while *BRCA2* carriers have a cumulative risk of approximately 29% [5]. Generally, germline GT has revealed PV in the *BRCA1* and *BRCA2* genes in up to 15% of women diagnosed with OC, highlighting a significant hereditary component [8–13]. Additionally, somatic PV in these same genes are identified in approximately 7% of cases, further emphasizing the importance of comprehensive molecular profiling in guiding personalized treatment strategies [6, 8]. Somatic mutation testing has emerged as a cornerstone of precision oncology in managing OC, offering critical insights into tumor biology and guiding personalized therapeutic strategies [6]. Somatic testing can also detect PV that can be of germline origin. Routine practice now favors somatic tumor testing in all advanced OC [6, 8, 14]. Historically, the widespread adoption of comprehensive tumor profiling was limited by therapeutic strategies, high sequencing costs and technological constraints [10, 13, 15]. Formalin-fixed paraffin-embedded (FFPE) tissue remains the most widely available and routinely archived material for molecular testing in OC [16]. At the same time, FFPE-derived DNA is often fragmented and chemically modified, which compromises the accuracy of downstream applications. However, the development of targeted next-generation sequencing (NGS) using comprehensive gene panels has provided a cost-effective and scalable solution [6, 17]. These advances have enabled FFPE-based NGS integration into routine clinical practice, supporting precision oncology in OC.

High-grade serous ovarian cancer (HGSOC) represents the most prevalent subtype of epithelial OC, accounting for the majority of cases and associated with poor prognosis due to its aggressive nature and frequent late-stage diagnosis [4, 18, 19]. Given the heterogeneous mutation spectrum in OC, broad tumor profiling panels can identify established driver genes and emerging tumor-specific biomarkers. These include homologous recombination deficiency (HRD) [6], microsatellite instability (MSI) [8], and tumor mutation burden (TMB) [20, 21], all of which can inform therapeutic decisions. Approximately 50% of HGSOC cases exhibit HRD [3, 6, 22]. HRD manifests through characteristic genomic alterations, including large-scale transitions, loss of heterozygosity, and telomeric allelic imbalance that reflect impaired homologous recombination repair beyond detectable PV in canonical HR genes. As such, HRD serves as a surrogate biomarker for predicting poly-ADP ribose polymerase (PARP) inhibitor sensitivity [6, 22, 23]. The OC treatment landscape has evolved with PARP inhibitors showing established clinical benefit, and emerging evidence suggests potential benefit from immunotherapies [6, 22, 24, 25]. Integration of tumor sequencing results with clinical parameters, such as chemotherapy response, progression-free survival, and tumor characteristics, can refine prognostic accuracy and support more optimized treatment selection [11]. It has been demonstrated that tumor DNA testing for *BRCA1*, *BRCA2*, and other relevant molecular markers provides additional value even when germline GT has already been performed, as tumor GT can uncover somatic alterations that are missed by germline analysis [8, 12]. This work provides the most comprehensive overview of actionable PV and biomarker profiles in Estonia, supporting the integration of precision medicine into routine oncology across the national healthcare system, and offering additional value through its combined somatic and germline testing perspective.

## Patients/material and methods

### Participants

The OC tumor samples underwent comprehensive genomic profiling at the Genetics and Personalized Medicine Clinic of Tartu University Hospital (TUH) between 2020 and 2024. As TUH is the only accredited center in Estonia performing OC molecular profiling, this represents a nationwide cohort, and the reported numbers reflect OC samples analyzed in the country during the study period. In total, 339 tumor samples were profiled, of which three were excluded due to insufficient DNA quality. Clinical data, including age at diagnosis, tumor cell fraction, histological subtype, and results of germline testing, were retrospectively obtained from test requisition forms completed by the ordering clinicians. Patients diagnosed with OC but not referred for molecular testing were not included, as molecular data were unavailable for these cases.

### OC GT approaches during the study period

**Figure 1:**
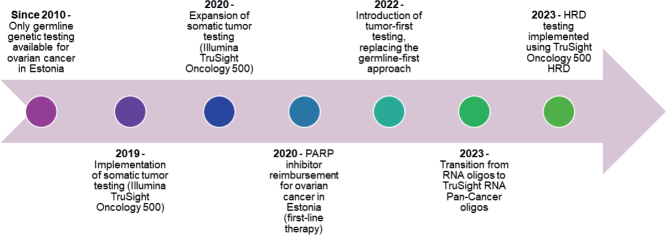
Timeline of ovarian cancer genomic testing and related clinical implementations in Estonia.

Until 2020, OC GT was primarily employed through germline-targeted testing approaches [30]. Somatic tumor testing was introduced in 2020 using TruSight Oncology 500 (523 genes; Illumina Inc.). Since 2022, OC testing has primarily followed a tumor-first GT strategy, with possible germline GT performed via Sanger seuqencing or targeted germline NGS panels. At the beginning of 2023, TSO500 RNA oligos were replaced with TruSight RNA Pan-Cancer oligos, and the add-on oligos of TruSight Oncology 500 HRD were introduced in July 2023 for HRD testing (Illumina, Inc.). Library preparation and sequencing were performed on the Illumina NextSeq 500 platform using 2×101bp paired-end reads, following the manufacturer’s protocols (Figure 1).

### Tumor next-generation sequencing data analysis and data interpretation

Data were analyzed using the Illumina TSO500 Local App software versions 2.0–2.2 and from 2023, Illumina Dragen V4 server with DRAGEN TruSight Oncology 500 Analysis Software v.2.1.1 to v.2.5.3 (Illumina, Inc.). The filtering and annotation of identified genetic variants were performed using bcftools and variant effect predictor (VEP) software. For variant annotation, databases such as OncoKB, cBioPortal, VarSome, COSMIC, Franklin, MyCancerGenome, dbSNP, gnomAd, and ClinVar were utilized, along with predictive tools for protein function assessment, including Sorting Intolerant From Tolerant (SIFT), PolyPhen2, Combined Annotation-Dependent Depletion (CADD), and PhyloP. Variant classification followed American College of Medical Genetics (ACMG) guidelines [26], and variants were described according to the HGVS Nomenclature [27]. Only class 4 and 5 variants, as classified according to ACMG/Association of Molecular Pathology (AMP) guidelines, were reported in the clinical workflow [26]. Cutoff values for genomic biomarkers were defined as follows: single-nucleotide variants (SNV) were reported at a variant allele frequency (VAF) ≥ 5%; gene amplifications were considered significant at ≥ 5 copies, the applied method does not differentiate between focal amplifications and larger-scale copy-number increases across the locus. TMB-HIGH was classified as high at ≥ 10 mutations per megabase (mut/Mb); MSI-HIGH was defined as ≥ 20% unstable MSI sites; and HRD was considered positive in HGSOC when % VAF was ≥ 30 and the HRD score was ≥ 42, based on the TruSight Oncology 500 HRD assay aligned with the Myriad MyChoice methodology.

## Results

### Cohort characterization

This study employed comprehensive molecular profiling in routine clinical practice to analyze the mutational landscape and actionable oncogenic variants in OC tumor samples in Estonia. A total of 339 tumor samples from distinct individuals were included. The number of additional material requests was not recorded. The mean age at diagnosis for OC patients was 62.4 ± 12.5 years, while the mean age at comprehensive tumor profiling was 63.5 ± 12.4 years. The number of tumor samples tested increased threefold over the years, from 38 samples in 2020 to 114 samples in 2024.

### Tumor characteristics

Most OC tumors were of primary origin, comprising 221 cases (65.2%), metastatic tumors accounted for 104 (30.7%) and unknown origin was reported in 14 samples (4.1%). HGSOC was the predominant histological subtype evaluated by pathologists, observed in 278 cases (82.0%), while low-grade serous ovarian cancer (LGSOC) represented 20 cases (5.9%). Other OC combined histologies accounted for 33 cases (9.7%), and an unknown histological type was reported in eight cases (2.4%). The mean pathologist‑estimated tumor fraction for OC samples was ~57.0% ± 24.0.

### Sequencing metrics

The average sequencing coverage across all samples was 1171.6x(± 362.0), reflecting a high sequencing depth. Furthermore, the proportion of bases covered at 100x averaged 97.9% (± 1.0%).

### Molecular findings in OC tumor samples

Among the 336 samples, we identified 529 single nucleotide PV. However, 14 samples harbored no PV. Among the detected PV, *TP53* was the most frequently altered gene, present in 79.5% of cases. *BRCA1* PV were the second most common (14.9%), followed by *KRAS* (6.0%), *BRCA2* (5.7%), and *PIK3CA* (5.1%). In the HGSOC subtype, the most prevalent PV were *TP53* 87.4% of cases, *BRCA1* 16.2% of cases and *BRCA2* (6.1%). In the other OC subtypes, the most prevalent PV were *TP53* (41.4%), followed by a markedly higher occurrence of *KRAS* (20.7%), *ARID1A* (17.2%), *PIK3CA* (13.8%), and *CTNNB1* (12.1%). The complete list of genes where PV was detected is shown Table 1. The most frequent *BRCA1* PV were c.5266dup, detected in 12 of 336 individuals (3.6%), and c.4035del, detected in 10 of 336 individuals (3.0%). All PV by HGVS are listed in the additional information file ‘allPVs’.

**Table 1:** Ovarian cancer histological types and the distribution of pathogenic/likely pathogenic genetic variants in tumor samples.

GENE	HGSOC (*n* = 278)	% of HGSOC (*n* = 278)	Other (*n* = 58)	% of other (*n* = 58)	Total	% of total 336
***TP53***	243	87.4	24	41.4	267	79.5
***BRCA1***	45	16.2	5	8.6	50	14.9
***KRAS***	8	2.9	12	20.7	20	6.0
***BRCA2***	17	6.1	2	3.4	19	5.7
***ARID1A***	7	2.5	10	17.2	17	5.1
***PIK3CA***	9	3.2	8	13.8	17	5.1
***NF1***	12	4.3	1	1.7	13	3.9
***CTNNB1***	3	1.1	7	12.1	10	3.0
***PTEN***	5	1.8	3	5.2	8	2.4
***RB1***	7	2.5	1	1.7	8	2.4
***BRAF***	2	0.7	5	8.6	7	2.1
***PIK3R1***	3	1.1	4	6.9	7	2.1
***BRIP1***	5	1.8	1	1.7	6	1.8
***PPP2R1A***	3	1.1	3	5.2	6	1.8
***ATM***	2	0.7	3	5.2	5	1.5
***CHEK2***	2	0.7	3	5.2	5	1.5
***FBXW7***	3	1.1	2	3.4	5	1.5
***CDKN2A***	1	0.4	3	5.2	4	1.2
***NRAS***	0	0.0	4	6.9	4	1.2
***PALB2***	4	1.4	0	0.0	4	1.2
***ERBB2***	1	0.4	2	3.4	3	0.9
***MSH2***	1	0.4	2	3.4	3	0.9
***NBN***	3	1.1	0	0.0	3	0.9
***APC***	0	0.0	2	3.4	2	0.6
***BAP1***	0	0.0	2	3.4	2	0.6
***BARD1***	2	0.7	0	0.0	2	0.6
***ERBB3***	0	0.0	2	3.4	2	0.6
***FGFR2***	1	0.4	1	1.7	2	0.6
***FOXL2***	0	0.0	2	3.4	2	0.6
***LZTR1***	2	0.7	0	0.0	2	0.6
***MSH6***	0	0.0	2	3.4	2	0.6
***NF2***	1	0.4	1	1.7	2	0.6
***TSC1***	2	0.7	0	0.0	2	0.6
***ARID1B***	0	0.0	1	1.7	1	0.3
***BLM***	1	0.4	0	0.0	1	0.3
***CDK12***	1	0.4	0	0.0	1	0.3
***CDKN1B***	0	0.0	1	1.7	1	0.3
***CHEK1***	1	0.4	0	0.0	1	0.3
***ESR1***	1	0.4	0	0.0	1	0.3
***KDM6A***	1	0.4	0	0.0	1	0.3
***MAP2K1***	1	0.4	0	0.0	1	0.3
***MAP3K1***	1	0.4	0	0.0	1	0.3
***MRE11***	1	0.4	0	0.0	1	0.3
***MUTYH***	1	0.4	0	0.0	1	0.3
***RAD54L***	1	0.4	0	0.0	1	0.3
***SDHA***	0	0.0	1	1.7	1	0.3
***SMARCB1***	1	0.4	0	0.0	1	0.3
***STK11***	0	0.0	1	1.7	1	0.3
***TERT***	0	0.0	1	1.7	1	0.3
***TSC2***	0	0.0	1	1.7	1	0.3
***VHL***	0	0.0	1	1.7	1	0.3
**TOTAL**	**405**		**124**		**529**	

Regarding the detected gene amplification events, *PIK3CA* was the most frequently amplified gene, observed in 34.2% of cases (Supplement table S1). Among the whole gene deletions identified, *STK11* was the most frequent, accounting for 5.4% of cases (Supplement table S2). In the studied cohort, we identified two AR-V7 splicing alterations. Among the identified gene fusions, the most prevalent involved the *ESR1* gene, accounting for eight events (2.4%) (Supplement table S3).

### Tumor biomarker results

Among the 336 samples evaluated for TMB, only 13 (3.9%) reported as TMB-HIGH. MSI analysis revealed a similarly low instability frequency, with MSI-HIGH status detected in nine (2.7%) cases. HRD analysis was implemented in 2023 as part of our comprehensive tumor testing regimen in clinical practice. HRD analysis was performed in 94 of HGSOC, and HRD+ was reported in 50 cases (53.2%) of HGSOC.

Actionable variants were evaluated using the OncoKB database, focusing on Level 1 and 2 evidence for OC. Among OC‑specific actionable alterations, 126 individuals (37.5%) carried at least one actionable variant. The most frequently altered actionable gene was *BRCA1* (14.6%), followed by *KRAS* (6.0%). Additional actionable findings are detailed in Table 2. When considering actionable biomarkers relevant to solid tumors more broadly, 56.3% of patients in our cohort had at least one targetable alteration (Supplement Table S4).

**Table 2:** Actionable variants identified in ovarian cancer tumor samples according to OncoKB Level 1 and Level 2 evidence in ovarian cancer.

Actionable oncogenic variants according to OncoKB Level 1 and 2			
Genes with PV	Number	% of OncoKB Level 1 and 2	% of 336
*BRCA1*	50	54.4	14.9
*KRAS*	20	21.7	6.0
*BRCA2*	19	20.7	5.7
*BRAF* (V600)	3	3.2	0.9
**TOTAL**	**92**	**100**	**27.5**
**Fusions**			
* NTRK3*	1	100	0.3
**TOTAL**	**1**	**100**	**0.3**
**Tumor biomarkers**			
* *TMB-HIGH	13	18.1	3.9
* *MSI-HIGH	9	12.5	2.7
* *HRD+	50	69.4	14.9
**TOTAL**	**72**	**100**	**21.4**
**Total number of ovarian cancer tumor samples with at least one actionable variant**	**126**	**100**	**37.5**

PV: pathogenic; HRD: homologous recombination deficiency; MSI: microsatellite instability; TMB: tumor mutation burden.

### Germline diagnostic strategies employed on OC individuals

Among the individuals whose tumors were included in the somatic testing cohort, 142 (41.9%) had also undergone germline GT. From the overall germline GT results, PV was identified in 32 out of 142 (22.5%) individuals. The most frequently affected gene was *BRCA1*, with 16 carriers (11.3%), followed by *BRCA2* in seven individuals (4.9%). The main *BRCA1* PVs were c.4035del and c.5266dup, accounting for 68.8% of all germline *BRCA1* PVs and 34.4% of all identified PVs. The primary *BRCA2* PV identified was c.8572C>T, accounting for 28.6% of all *BRCA2* germline PV. Additional germline PV were detected in *CHEK2* in three patients (2.1%) and in *BRIP1* in two patients (1.4%). Among the reported germline PV, one (3.2%) was not detected in the corresponding tumor tissue, a duplication in the *RAD51C* gene, encompassing exons 6 and 7. No germline PV were identified in 110 individuals (77.5%). All detected germline PV are shown in Supplement information ‘AllPVs’.

## Discussion and conclusion

This nationwide retrospective analysis of 339 OC tumor samples demonstrates the feasibility and clinical value of implementing comprehensive molecular profiling within routine diagnostics in Estonia. The number of tested tumors increased steadily from 2020 to 2024, representing a threefold increase and illustrating the adoption of precision oncology practices across Estonian oncology centers. The mean age at OC diagnosis in our cohort was 62.4 years comparable to or slightly higher than onset ages reported in the USA (63.0), Croatia (59.0), and Tunisia (52.4) [18, 28, 29]. In Estonia prior to 2020, clinical practice relied primarily on germline GT. Somatic tumor profiling was introduced later and was ordered selectively for previously germline-tested patients when additional tumor-level information was needed for therapeutic decision-making. Following the reimbursement of PARP inhibitors such as olaparib in 2020 in first-line maintenance treatment, Estonia introduced a tumor‑first GT approach in 2022, with optional germline testing, alongside a marked increase in testing volumes (Figure 1). However, in our cohort, the median time from diagnosis to somatic testing was 1.1 years, whereas the median time to germline testing was substantially longer, 3.3 years [30]. This indicates that the implementation of somatic testing has, in fact, improved the timeliness of molecular evaluation in Estonia, as patients are now receiving at least somatic testing earlier than they historically received germline testing.

Recent Nordic and European initiatives further highlight the growing momentum toward precision cancer medicine. Large-scale collaborative programs such as PCM4EU and PRIME-ROSE have demonstrated the importance of coordinated precision medicine implementation across Europe to ensure equitable access to molecularly guided therapies, a development in which Estonia is also actively participating. Similar national initiatives in Norway and Finland show how structured precision medicine programs can rapidly increase patient inclusion, improve diagnostic workflows, and generate real-world evidence to support clinical decision-making. Nordic experts also emphasize that while precision medicine holds great promise, its successful integration into routine oncology requires sustained investment, interdisciplinary collaboration, and continuous evaluation of clinical impact [31].

Only three tumor samples (0.9%) failed quality control due to insufficient genomic material. Information regarding additional requests for FFPE material for nucleic acid extraction was not available. This failure rate is notably lower than those reported in previous studies, including 2.9% in a Japanese cohort and 6.1% in a multicenter study by Jantus-Lewintre et al., conducted across Austria, the Netherlands, Italy, France, and Spain [32, 33]. Centralized molecular profiling at TUH ensured high technical reliability, achieving a mean sequencing depth of 1171× and 97.9% of bases covered at ≥ 100× across all samples. These parameters enabled robust detection of somatic variants in FFPE-derived tumors and surpassed sequencing coverage reported in studies from Greece (500×), Croatia (500×; ≥ 100× > 99%), and a multicenter study by Jantus-Lewintre et al. (500×) [11, 28, 33]. This high level of sequencing coverage is critical for ensuring accurate variant calling in clinical cancer genomic profiling workflows.

*TP53* PV are the hallmark alteration of HGSOC, present in over half of cases and including rare in-frame insertions, deletions, and frameshift mutations [28, 29, 34, 35]. Their prognostic significance remains unclear, though *in vitro* data suggest potential effects on PARP-inhibitor response [36]. In mucinous OC and LGSOC, PV in *KRAS*, *BRAF*, and *NRAS* are key drivers that converge on aberrant MAPK-pathway activation. Because *KRAS*-driven MAPK signaling underlies the pathogenesis of these subtypes, MEK-targeted therapy, as well as emerging EGFR-directed and combined MAPK/PI3K-pathway strategies, represent the most rational therapeutic approaches [34, 35, 37–39].

*BRCA1* and *BRCA2* PV occurred in 14.9% and 5.7% of tumors, which is well within the expected range for combined somatic and germline BRCA-associated OC (~15–20%) [12, 29]. Both previously known *BRCA1* main PV in Estonia (c.5266dup and c.4035del) and the *BRCA2* c.8572C>T PV were the most common alterations, mirroring the germline *BRCA1/2* spectrum previously identified in the Estonian Biobank, regional cohorts [28, 40–43], and published data from the Estonian OC cohort by Tooming et al., where c.5266dup and c.4035del germline PV were found in 11.7% of 759 OC patients [30]. Importantly, in one patient, germline PV was not detected in the corresponding tumor, demonstrating that tumor-only analysis may fail to identify a subset of heritable variants. Guidelines from the European Society for Medical Oncology and the American Society of Clinical Oncology indicate that tumor-only sequencing may miss approximately 5% of germline *BRCA1/2* PV [8, 14], emphasizing the clinical importance of paired tumor-germline testing for accurate risk assessment and cascade testing.

Biomarker analysis revealed low frequencies of TMB-HIGH (3.9%) and MSI-HIGH tumors (2.7%), consistent with reported rates in OC by Morand et al. (TMB-HIGH 3.6%) and Landen et al. (TMB-HIGH 3%; MSI-HIGH 0.3%) [24, 44]. As expected, HRD+ was detected in 53.2% of HGSOC, aligning with the ~50% prevalence reported in the literature [3, 6, 22]. However, HRD evaluation is technically complex, requiring a sufficiently high pathologist-assessed tumor cell fraction (30%) and reliable estimation for genomic instability metrics. As a result, only samples that fulfilled these criteria were included in the HRD analysis. Conversely, since HRD testing became part of routine tumor profiling in 2023, it is expected to play an increasingly important role in guiding treatment decisions for OC patients in Estonia.

Actionable variants defined by OncoKB Level 1& 2 in OC evidence were identified in 37.5% of patients, and this proportion is consistent with other national OC genomic profiling studies (~> 20%) [11, 13, 28, 29, 34], underscoring the clinical value of comprehensive tumor sequencing. The strongest evidence exists for *BRCA1/2* and HRD‑associated alterations, which underpin the approved use of PARP inhibitors in OC. U.S. Food and Drug Administration (FDA)‑approved PARP inhibitor options are now largely restricted to the maintenance setting [9, 13, 22]. These indications closely align with European Medicines Agency (EMA) practice and remain most applicable to the HGSOC subgroup, where HRD and *BRCA* PV are concentrated [45]. Beyond HRD, our results identified additional, potentially druggable alterations. *KRAS* mutations, which are particularly enriched in non-HGSOC tumors such as LGSOC [46], have recently gained direct therapeutic relevance following the accelerated FDA approval (May 2025) of the avutometinib + defactinib combination for *KRAS*-mutated recurrent LGSOC. As these agents have not yet received EMA approval, patients in Europe currently require access through clinical trial participation or structured drug-repurposing pathways such as DRUP-type programs [15, 47]. Conversely, *NTRK3* gene fusions are directly actionable through the tumor-agnostic FDA and EMA approvals of larotrectinib and entrectinib, making these alterations immediately clinically relevant for patients identified with such fusions. In addition, several variants identified in *ERBB2*, *MET*, *FGFR2*, and components of the PI3K/mTOR pathway represent additional potentially targetable alterations. Although these biomarkers have received FDA and EMA approvals for targeted therapies in other solid tumors, they are not yet specifically approved for OC. Nevertheless, clinical trials, such as the DESTINY-PanTumor02 study, are actively evaluating their therapeutic relevance in this setting [48]. These alterations may provide eligibility for tumor‑agnostic basket trials evaluating targeted strategies across cancer types. In such cases, patients may also qualify for precision‑oncology trials or compassionate‑use and/or drug‑repurposing programs, including DRUP‑type frameworks [15, 47].

The absence of longitudinal clinical outcome data limited our ability to assess the clinical relevance of the detected alterations. Additionally, germline testing was inconsistently performed, some patients had undergone germline analysis prior to somatic testing, whereas others had not undergone germline testing at all constraining our capacity to systematically evaluate germline somatic relationships. Future studies should integrate comprehensive genomic profiling with complete clinical datasets, including standardized germline testing, to enable robust outcome-based analyses and further advance precision oncology.

Collectively, these findings demonstrate that integrating comprehensive tumor genomic profiling with germline GT substantially enhances diagnostic yield and therapeutic precision for OC patients in Estonia. The high prevalence of actionable biomarkers, particularly PV *BRCA1/2* and HRD, supports the continued expansion of molecular diagnostics and integrative precision oncology frameworks. Although paired tumor-germline testing represents the optimal GT strategy, its routine adoption may be challenging due to cost or logistical constraints [49]. Expanding access where feasible, integrating HRD assessment, and improving clinical data linkage remain key to advancing OC precision care in Estonia.

## Supplementary Material





## Data Availability

The genomic data generated in this study originate from accredited clinical diagnostic testing at TUH. Due to institutional policy, patient consent restrictions, and national data protection regulations, raw sequencing files and individual level genetic data cannot be deposited in public repositories. De-identified aggregate data supporting the findings of this study are included within the article and its supplementary materials. Access to controlled level clinical genomic data may be granted on reasonable request for research purposes that comply with applicable regulations. Requests should be directed to Mikk Tooming (mikk.tooming@kliinikum.ee).

## References

[1] WHO. WHO, cancer today [Internet]. 2026 [cited 2026 Feb 21]. Available from: https://gco.iarc.who.int/today/

[2] Tervisestatistika ja terviseuuringute andmebaas. PK10: Pahaloomuliste kasvajate esmasjuhud paikme, soo ja vanuserühma järgi [Internet]. [cited 2025 Apr 14]. Available from: http://statistika.tai.ee/pxweb/et/Andmebaas/Andmebaas__02Haigestumus__04PahaloomulisedKasvajad/PK10.px/

[3] Bell D, Berchuck A, Birrer M, Chien J, Cramer DW, Dao F, et al. Integrated genomic analyses of ovarian carcinoma. Nature. 2011;474(7353):609–15. 10.1038/nature10166 21720365 PMC3163504

[4] Shih IM, Wang Y, Wang TL. The origin of ovarian cancer species and precancerous landscape. Am J Pathol. 2021;191(1):26–39. 10.1016/j.ajpath.2020.09.006 33011111 PMC7786078

[5] Daly MB, Pal T, Maxwell KN, Churpek J, Kohlmann W, AlHilli Z, et al. NCCN guidelines® insights: genetic/familial high-risk assessment: breast, ovarian, and pancreatic, version 3.2026. J Natl Compr Canc Netw [Internet]. 2026;(v3.2026). [cited 2026 Feb 2]. Available from: https://www.nccn.org/professionals/physician_gls/pdf/genetics_bopp.pdf

[6] Gressel GM, Frey MK, Norquist B, Senter L, Blank SV, Urban RR. Germline and somatic testing for ovarian cancer: an SGO clinical practice statement. Gynecol Oncol. 2024;181:170–8. 10.1016/j.ygyno.2023.12.010 38215513

[7] Casaubon JT, Kashyap S, Regan JP. BRCA1 and BRCA2 mutations [Internet]. Treasure Island, FL: StatPearls Publishing; 2025 [cited 2026 Feb 21]. Available from: http://www.ncbi.nlm.nih.gov/books/NBK470239/29262038

[8] Konstantinopoulos PA, Norquist B, Lacchetti C, Armstrong D, Grisham RN, Goodfellow PJ, et al. Germline and somatic tumor testing in epithelial ovarian cancer: ASCO guideline. J Clin Oncol. 2020;38(11):1222–45. 10.1200/JCO.19.02960 31986064 PMC8842911

[9] Barbosa A, Pinto P, Peixoto A, Guerra J, Pinto C, Santos C, et al. Gene panel tumor testing in ovarian cancer patients significantly increases the yield of clinically actionable germline variants beyond BRCA1/BRCA2. Cancers. 2020;12(10):2834. 10.3390/cancers12102834 33008098 PMC7650720

[10] Jang J, Kim Y, Kim JH, Cho SM, Lee KA. Cost-effectiveness analysis of germline and somatic BRCA testing in patients with advanced ovarian cancer. Ann Lab Med. 2023;43(1):73–81. 10.3343/alm.2023.43.1.73 36045059 PMC9467835

[11] Andrikopoulou A, Zografos E, Apostolidou K, Kyriazoglou A, Papatheodoridi AM, Kaparelou M, et al. Germline and somatic variants in ovarian carcinoma: a next-generation sequencing (NGS) analysis. Front Oncol. 2022;12:1030786. 10.3389/fonc.2022.1030786 36531003 PMC9754718

[12] Frugtniet B, Morgan S, Murray A, Palmer-Smith S, White R, Jones R, et al. The detection of germline and somatic BRCA1/2 genetic variants through parallel testing of patients with high-grade serous ovarian cancer: a national retrospective audit. BJOG. 2022;129(3):433–42. 10.1111/1471-0528.16975 34657373 PMC9298909

[13] Vos JR, Fakkert IE, de Hullu JA, van Altena AM, Sie AS, Ouchene H, et al. Universal tumor DNA BRCA1/2 testing of ovarian cancer: prescreening PARPi treatment and genetic predisposition. J Natl Cancer Inst. 2020;112(2):161–9. 10.1093/jnci/djz080 31076742 PMC7019087

[14] Mosele MF, Westphalen CB, Stenzinger A, Barlesi F, Bayle A, Bièche I, et al. Recommendations for the use of next-generation sequencing (NGS) for patients with advanced cancer in 2024: a report from the ESMO Precision Medicine Working Group. Ann Oncol. 2024;35(7):588–606. 10.1016/j.annonc.2024.04.005 38834388

[15] Tavares V, Marques IS, de Melo IG, Assis J, Pereira D, Medeiros R. Paradigm shift: a comprehensive review of ovarian cancer management in an era of advancements. Int J Mol Sci. 2024;25(3):1845. 10.3390/ijms25031845 38339123 PMC10856127

[16] Carrick DM, Mehaffey MG, Sachs MC, Altekruse S, Camalier C, Chuaqui R, et al. Robustness of next generation sequencing on older formalin-fixed paraffin-embedded tissue. PLoS One. 2015;10(7):e0127353. 10.1371/journal.pone.0127353 26222067 PMC4519244

[17] Edsjö A, Russnes HG, Lehtiö J, Tamborero D, Hovig E, Stenzinger A, et al. High-throughput molecular assays for inclusion in personalised oncology trials – state-of-the-art and beyond. J Intern Med. 2024;295(6):785–803. 10.1111/joim.13785 38698538

[18] Caruso G, Weroha SJ, Cliby W. Ovarian cancer: a review. JAMA. 2025;334(14):1278–91. 10.1001/jama.2025.9495 40690248

[19] Giro A, Herrmann T, Bauer A, Pinard C, Godiveau M, Passildas J, et al. Predictive and prognostic factors in epithelial ovarian cancer: a review. Indian J Gynecol Oncolog. 2025;23(1):45. 10.1007/s40944-025-00964-8

[20] Nero C, Ciccarone F, Pietragalla A, Duranti S, Daniele G, Salutari V, et al. Ovarian cancer treatments strategy: focus on PARP inhibitors and immune check point inhibitors. Cancers. 2021;13(6):1298. 10.3390/cancers13061298 33803954 PMC7999042

[21] Cui M, Xia Q, Zhang X, Yan W, Meng D, Xie S, et al. Development and validation of a tumor mutation burden-related immune prognostic signature for ovarian cancers. Front Genet. 2022;12:688207. 10.3389/fgene.2021.688207 35087563 PMC8787320

[22] O’Malley DM, Krivak TC, Kabil N, Munley J, Moore KN. PARP inhibitors in ovarian cancer: a review. Target Oncol. 2023;18(4):471–503. 10.1007/s11523-023-00970-w 37268756 PMC10344972

[23] Watkins JA, Irshad S, Grigoriadis A, Tutt AN. Genomic scars as biomarkers of homologous recombination deficiency and drug response in breast and ovarian cancers. Breast Canc Res. 2014;16(3):211. 10.1186/bcr3670 PMC4053155 25093514

[24] Morand S, Devanaboyina M, Staats H, Stanbery L, Nemunaitis J. Ovarian cancer immunotherapy and personalized medicine. Int J Mol Sci. 2021;22(12):6532. 10.3390/ijms22126532 34207103 PMC8234871

[25] Colombo N, Coleman RL, Wu X, Köse F, Wenham RM, Sebastianelli A, et al. 2022-RA-657-ESGO ENGOT-ov65/KEYNOTE-B96: phase 3, randomized, double-blind study of pembrolizumab versus placebo plus paclitaxel with optional bevacizumab for platinum-resistant recurrent ovarian cancer. Int J Gynecol Cancer. 2022;32:A251. 10.1136/ijgc-2022-ESGO.538

[26] Richards S, Aziz N, Bale S, Bick D, Das S, Gastier-Foster J, et al. Standards and guidelines for the interpretation of sequence variants: a joint consensus recommendation of the American College of Medical Genetics and Genomics and the Association for Molecular Pathology. Genet Med. 2015;17(5):405–23. 10.1038/gim.2015.30 25741868 PMC4544753

[27] den Dunnen JT, Dalgleish R, Maglott DR, Hart RK, Greenblatt MS, McGowan-Jordan J, et al. HGVS recommendations for the description of sequence variants: 2016 update. Hum Mutat. 2016;37(6):564–9. 10.1002/humu.22981 26931183

[28] Čerina D, Matković V, Katić K, Belac Lovasić I, Šeparović R, Canjko I, et al. Comprehensive genomic profiling in the management of ovarian cancer – national results from croatia. J Pers Med. 2022;12(7):1176. 10.3390/jpm12071176 35887672 PMC9322425

[29] Ammous-Boukhris N, Abdelmaksoud-Dammak R, Ben Kridis W, Ben-Ayed-Guerfali D, Shtaiwi Abed A, Guidara S, et al. Germline and somatic mutational variants of Tunisian high grade serous ovarian cancer identified by next-generation sequencing. BMC Cancer. 2025;25:1542. 10.1186/s12885-025-14989-x 41068641 PMC12513094

[30] Tooming M, Toome K, Rekker K, Roht L, Laidre P, Fjodorova O, et al. Exploring the hereditary genetic mutational landscape of breast and ovarian cancer in Estonia. Sci Rep. 2026;16(1):13373. Available from: https://www.nature.com/articles/s41598-026-43459-y doi: https://doi.org/10.1038/s41598-026-43459-y 41826624 10.1038/s41598-026-43459-y PMC13106779

[31] Nilbert M. The promises of precision medicine – voices from the Nordics. Acta Oncol. 2025;64:775–7. 10.2340/1651-226X.2025.43987 40500958 PMC12175172

[32] Imoto K, Yamamoto H, Ohkawa C, Shimada N, Ikuzawa R, Takeda H, et al. An approach for improvement of the accuracy of cancer gene panel testing. Int J Clin Oncol. 2024;29(5):571–81. 10.1007/s10147-024-02483-6 38472663

[33] Jantus-Lewintre E, Rappa A, Ruano D, van Egmond D, Gallach S, Gozuyasli D, et al. Multicenter in-house evaluation of an amplicon-based next−generation sequencing panel for comprehensive molecular profiling. Mol Diagn Ther. 2025;29(2):249–61. 10.1007/s40291-024-00766-2 39798063 PMC11860996

[34] Fieuws C, Van der Meulen J, Proesmans K, De Jaeghere EA, Loontiens S, Van Dorpe J, et al. Identification of potentially actionable genetic variants in epithelial ovarian cancer: a retrospective cohort study. npj Precis Onc. 2024;8(1):1–9. 10.1038/s41698-024-00565-2 PMC10959961 38519644

[35] Veneziani AC, Gonzalez-Ochoa E, Alqaisi H, Madariaga A, Bhat G, Rouzbahman M, et al. Heterogeneity and treatment landscape of ovarian carcinoma. Nat Rev Clin Oncol. 2023;20(12):820–42. 10.1038/s41571-023-00819-1 37783747

[36] Smith LE, Padilla JL, Licor A, Steinkamp MP, Lagutina IV, Guo Y, et al. Novel p53 reactivators that are synergistic with olaparib for the treatment of gynecologic cancers with mutant p53. Transl Oncol. 2025;61:102522. 10.1016/j.tranon.2025.102522 40915175 PMC12450570

[37] El-Bahrawy M. Ovarian cancer pathology. In: Farghaly SA, editor. Advances in diagnosis and management of ovarian cancer. Cham: Springer International Publishing; 2022. p. 57–85. 10.1007/978-3-031-09169-8_5

[38] Kelliher L, Yoeli-Bik R, Schweizer L, Lengyel E. Molecular changes driving low-grade serous ovarian cancer and implications for treatment. Int J Gynecol Cancer. 2024;34(10):1630–8. 10.1136/ijgc-2024-005305 38950921 PMC11503204

[39] Shim YS, Kim JH, Seo S-S, Kang S, Park SY, Lim MC. Landscape of genomic alterations and clinical outcomes in low-grade serous ovarian cancer in Korea. Gynecol Oncol Rep. 2025;61:101934. 10.1016/j.gore.2025.101934 40977829 PMC12447911

[40] Koczkowska M, Zuk M, Gorczynski A, Ratajska M, Lewandowska M, Biernat W, et al. Detection of somatic BRCA1/2 mutations in ovarian cancer – next‐generation sequencing analysis of 100 cases. Cancer Med. 2016;5(7):1640–6. 10.1002/cam4.748 27167707 PMC4867663

[41] Leitsalu L, Palover M, Sikka TT, Reigo A, Kals M, Pärn K, et al. Genotype-first approach to the detection of hereditary breast and ovarian cancer risk, and effects of risk disclosure to biobank participants. Eur J Hum Genet. 2021;29(3):471–81. 10.1038/s41431-020-00760-2 33230308 PMC7940387

[42] Jürgens H, Roht L, Leitsalu L, Nõukas M, Palover M, Nikopensius T, et al. Precise, genotype-first breast cancer prevention: experience with transferring monogenic findings from a population biobank to the clinical setting. Front Genet. 2022;13:881100. 10.3389/fgene.2022.881100 35938029 PMC9355130

[43] Tamboom K, Kaasik K, Aršavskaja J, Tekkel M, Lilleorg A, Padrik P, et al. BRCA1 mutations in women with familial or early-onset breast cancer and BRCA2 mutations in familial cancer in Estonia. Hered Cancer Clin Pract. 2010;8(1):4. 10.1186/1897-4287-8-4 20380699 PMC2867795

[44] Landen CN, Molinero L, Hamidi H, Sehouli J, Miller A, Moore KN, et al. Influence of genomic landscape on cancer immunotherapy for newly diagnosed ovarian cancer: biomarker analyses from the IMagyn050 randomized clinical trial. Clin Cancer Res. 2023;29(9):1698–707. 10.1158/1078-0432.CCR-22-2032 36595569 PMC10150250

[45] European Medicines Agency (EMA). European Medicines Agency (EMA) – olaparib [Internet]. 2026 [cited 2026 Mar 22]. Available from: https://www.ema.europa.eu/en/medicines/human/EPAR/lynparza

[46] Peng Y, Yang Q. Targeting KRAS in gynecological malignancies. FASEB J. 2024;38(19):e70089. 10.1096/fj.202401734R 39377766

[47] Villegas-Vazquez EY, Marín-Carrasco FP, Reyes-Hernández OD, Báez-González AS, Bustamante-Montes LP, Padilla-Benavides T, et al. Revolutionizing ovarian cancer therapy by drug repositioning for accelerated and cost-effective treatments. Front Oncol. 2025;14:1514120. 10.3389/fonc.2024.1514120 39876896 PMC11772297

[48] Meric-Bernstam F, Makker V, Oaknin A, Oh DY, Banerjee S, González-Martín A, et al. Efficacy and safety of trastuzumab deruxtecan in patients with HER2-expressing solid tumors: primary results from the DESTINY-PanTumor02 phase II trial. J Clin Oncol. 2024;42(1):47–58. 10.1200/JCO.23.02005 37870536 PMC10730032

[49] Kuzbari Z, Bandlamudi C, Loveday C, Garrett A, Mehine M, George A, et al. Germline-focused analysis of tumour-detected variants in 49,264 cancer patients: ESMO Precision Medicine Working Group recommendations. Ann Oncol. 2023;34(3):215–27. 10.1016/j.annonc.2022.12.003 36529447

