# Supplementary material for: Exploring the somatic mutational landscape of ovarian cancer in Estonia
Source: Acta Oncol. 2026 May 18;65:45612. doi: 10.2340/1651-226X.2026.45612 (PMC13192237; doi:10.2340/1651-226X.2026.45612)
Supplement: Supplementary file 1 [file AO-65-45612-s1.pdf]

Supplementary material has been published as submitted. It has not been copyedited, or typeset by Acta Oncologica

Supplement table S1. Reported gene amplifications from ovarian cancer tumours

Gene (amplification)	Count (n=1.022)	% of all amplifications (n=1.022)	% of 336
PIK3CA	115	11.3%	34.2%
MYC	83	8.1%	24.7%
ALK	77	7.5%	22.9%
EGFR	67	6.6%	19.9%
CCNE1	56	5.5%	16.7%
KRAS	43	4.2%	12.8%
PIK3CB	35	3.4%	10.4%
FGF23	34	3.3%	10.1%
MYCL	33	3.2%	9.8%
FGFR1	29	2.8%	8.6%
AKT2	28	2.7%	8.3%
MET	27	2.6%	8.0%
BRAF	26	2.5%	7.7%
AKT3	23	2.3%	6.8%
CCND1	23	2.3%	6.8%
FGFR2	19	1.9%	5.7%
CCND3	18	1.8%	5.4%
MDM4	18	1.8%	5.4%
MYCN	18	1.8%	5.4%
NTRK1	17	1.7%	5.1%
ERBB2	16	1.6%	4.8%
ERBB3	15	1.5%	4.5%
JAK2	15	1.5%	4.5%
CDK6	13	1.3%	3.9%
FGF19	13	1.3%	3.9%
BRD4	12	1.2%	3.6%
CDK4	11	1.1%	3.3%
FGFR4	10	1.0%	3.0%
MDM2	10	1.0%	3.0%
DDR2	9	0.9%	2.7%
FGF3	9	0.9%	2.7%
FGF4	9	0.9%	2.7%
AKT1	8	0.8%	2.4%
CCND2	7	0.7%	2.1%
KIT	7	0.7%	2.1%
PDGFRA	7	0.7%	2.1%
FGF1	6	0.6%	1.8%
RICTOR	6	0.6%	1.8%
FGFR3	5	0.5%	1.5%
HRAS	5	0.5%	1.5%
JUN	5	0.5%	1.5%
CD274	4	0.4%	1.2%
FGF8	4	0.4%	1.2%
ROS1	4	0.4%	1.2%
FGF7	3	0.3%	0.9%
MITF	3	0.3%	0.9%
CRLF2	2	0.2%	0.6%

EZH2	2	0.2%	0.6%
FGF2	2	0.2%	0.6%
FGF9	2	0.2%	0.6%
KDR	2	0.2%	0.6%
RAF1	2	0.2%	0.6%
AR	1	0.1%	0.3%
ETV1	1	0.1%	0.3%
MAPK1	1	0.1%	0.3%
MYB	1	0.1%	0.3%
MYCL1	1	0.1%	0.3%

Supplement table S2. Reported gene deletions from ovarian cancer tumours

Gene (deletion)	count (n=129)	% from all deletions (n=129)	% of 336 tumors
STK11	18	14.0%	5.4%
NF1	12	9.3%	3.6%
CDH1	10	7.8%	3.0%
ATRX	9	7.0%	2.7%
KDM6A	9	7.0%	2.7%
NF2	8	6.2%	2.4%
CDK12	7	5.4%	2.1%
IKZF1	7	5.4%	2.1%
DICER1	6	4.7%	1.8%
ARID1A	5	3.9%	1.5%
SMAD4	5	3.9%	1.5%
ATM	3	2.3%	0.9%
CDKN2B	3	2.3%	0.9%
PAX5	3	2.3%	0.9%
SMARCA4	3	2.3%	0.9%
BARD1	2	1.6%	0.6%
BRCA1	2	1.6%	0.6%
BRCA2	2	1.6%	0.6%
CREBBP	2	1.6%	0.6%
FBXW7	2	1.6%	0.6%
SMARCB1	2	1.6%	0.6%
TP53	2	1.6%	0.6%
VHL	2	1.6%	0.6%
CDKN2A	1	0.8%	0.3%
FH	1	0.8%	0.3%
MLH1	1	0.8%	0.3%
PTEN	1	0.8%	0.3%
SETD2	1	0.8%	0.3%

Supplement Table S3. Reported gene fusions from ovarian cancer tumours

Gene fusions	Count (n=18)	% of all fusions (n=18)	% of 336
ESR1::CCDC170	4	22.2%	1.2%
ESR1::C6orf211	2	11.1%	0.6%
FIP1L1::PDGFRA	2	11.1%	0.6%
ESR1::ERBB2	2	11.1%	0.6%
BRAF::NDUFB2	1	5.6%	0.3%
CTTN::ERBB2	1	5.6%	0.3%
EWSR1::BRMS1L	1	5.6%	0.3%
FGFR2::SEC23IP	1	5.6%	0.3%
FGFR2::TACC2	1	5.6%	0.3%
IQGAP1::NTRK3	1	5.6%	0.3%
KANSL1L::ERBB4	1	5.6%	0.3%
KMT2A::BACE1	1	5.6%	0.3%
KMT2A::UBE4A	1	5.6%	0.3%
MAP3K2::ETV4	1	5.6%	0.3%
PRRC2B::ABL1	1	5.6%	0.3%
RCL1::JAK2	1	5.6%	0.3%
SEC16A::NOTCH1	1	5.6%	0.3%
ZMIZ1::BRAF	1	5.6%	0.3%

Supplement table S4. Reported actionable variants in solid tumors according to OncoKB in the cohort

Actionable variants in solid tumors			
**Genes with PV**	**Number**	**% of PV OncoKB in solid tumors**	**% of 336**
BRCA1	50	28.9	14.9
KRAS	20	11.6	6.0
BRCA2	19	11.0	5.7
PIK3CA	17	9.8	5.1
NF1	13	7.5	3.9
PTEN	8	4.6	2.4
BRIP1	6	3.5	1.8
CHEK2	5	2.9	1.5
ATM	5	2.9	1.5
NRAS	4	2.3	1.2
PALB2	4	2.3	1.2
BRAF (V600)	3	1.7	0.9
ERBB2	3	1.7	0.9
BARD1	2	1.2	0.6
FGFR2	2	1.2	0.6
NBN	3	1.7	0.9
TSC1	2	1.2	0.6
CDK12	1	0.6	0.3
CHEK1	1	0.6	0.3
ESR1	1	0.6	0.3
MRE11	1	0.6	0.3
RAD54L	1	0.6	0.3
TSC2	1	0.6	0.3
MAP2K1	1	0.6	0.3
**TOTAL**	**173**	**100%**	**51.5%**
**Amplifications**			
MET	27	62.8%	8.0%
ERBB2	16	37.2%	4.8%
**TOTAL**	**43**	**100%**	**12.8%**
**Deletions**			
SMARCB1	2	100%	0.6%
**TOTAL**	**2**	**100%**	**0.6%**
**Fusions**			
BRAF	2	40.0%	0.6%
FGFR2	2	40.0%	0.6%
NTRK3	1	20.0%	0.3%
**TOTAL**	**5**	**100%**	**1.2%**
**Tumor biomarkers**			
TMB-HIGH	13	18.1%	3.9%
MSI-HIGH	9	12.5%	2.7%
HRD+	50	69.4%	14.9%

**TOTAL**	**72**	**100%**	**21.4%**
**Total number of ovarian cancer tumor samples with at least one actionable variant**	**189**	**100%**	**56.3%**
